# Anxiety and Depression in Tension-Type Headache: A Population-Based Study

**DOI:** 10.1371/journal.pone.0165316

**Published:** 2016-10-26

**Authors:** Tae-Jin Song, Soo-Jin Cho, Won-Joo Kim, Kwang Ik Yang, Chang-Ho Yun, Min Kyung Chu

**Affiliations:** 1 Department of Neurology, Ewha Womans University School of Medicine, Seoul, Korea; 2 Department of Neurology, Yonsei University, College of Medicine, Seoul, Korea; 3 Department of Neurology, Dongtan Sacred Heart Hospital, Hallym University College of Medicine, Hwaseong, Korea; 4 Department of Neurology, Gangnam Severance Hospital, Yonsei University, College of Medicine, Seoul, Korea; 5 Department of Neurology, Soonchunhyang University College of Medicine, Cheonan Hospital, Cheonan, Korea; 6 Clinical Neuroscience Center, Department of Neurology, Seoul National University Bundang Hospital, Seongnam, Korea; 7 Department of Neurology, Kangnam Sacred Heart Hospital, Hallym University College of Medicine, Seoul, Korea; Taipei Veterans General Hospital, TAIWAN

## Abstract

Although tension-type headache (TTH) is a frequent type of headache disorder and imposes a significant burden, there is scant information about the prevalence and impact of comorbid anxiety and depression among individuals with TTH. We investigated the prevalence and clinical impact of anxiety and depression among patients with TTH in the general population. We recruited Korean participants aged 19–69 years using a two-stage clustered random sampling method. To identify the presence of headache type, anxiety, and depression, we used a semi-structured interview using certain questionnaires. To assess the level of anxiety and depression, we used the Goldberg Anxiety Scale and Patient Health Questionnaire-9, respectively. Among 2,695 participants, 570 people (21.2%) had TTH during previous 1 year. In participants with TTH, the prevalence of anxiety (9.5% vs. 5.3%, *p* = 0.001) and depression (4.2% vs. 1.8%, *p* = 0.001) was significantly higher than that of non-headache participants. The prevalence of anxiety among TTH participants with >15 attacks per month [21.4%, odds ratio (OR): 4.0] and 1–14 attacks per month (13.1%, OR: 2.2) was higher than that in those with <1 attack per month (6.4%), however this tendency was not observed in participants with depression. Visual Analogue Scale (VAS) score [median 5.0 vs. 4.0, *p* = 0.010] and Headache Impact Test-6 (HIT-6) score [median 45.5 vs. 42.0, *p* < 0.001] were significantly higher among those with anxiety. Furthermore, VAS scores [median 5.0 vs. 4.0, *p* = 0.010] and HIT-6 scores [median 45.5 vs. 42.0, *p* = 0.027] were also significantly higher among TTH patients with depression than among those without depression. In conclusion, anxiety and depression were more prevalent in participants with TTH than in non-headache participants. These two conditions were associated with an exacerbation of headache symptoms in individuals with TTH.

## Introduction

Tension-type headache (TTH) is the most common type of headache and the third most common disorder in the world [1]. It has been considered as a non-serious disorder owing to its mild symptoms in most cases [2]. However, some TTH patients suffer from frequent or severe headache, and experience disability at work, school, or house [1]. Because of its high prevalence, TTH is a major health problem with huge socioeconomic burden [3]. Thus, identification of the associated factors of TTH may be an important public health issue.

Some individuals with TTH presents with comorbid psychiatric conditions. Among psychiatric illness, anxiety and depression were more frequently associated with TTH compared to non-headache participants [4, 5]. Moreover, previous studies reported that there is no significant difference between patients with TTH and patients with migraine (frequently associated with anxiety or depression) [6, 7] regarding psychiatric comorbidity [8–10]. In addition, anxiety and depression significantly affect sufferers’ quality of life and added a significant disability in patients with TTH [11].

Nevertheless, studies regarding the prevalence and clinical impact of anxiety and depression among individuals with TTH, particularly in general population, have rarely been reported. The Korean Headache Sleep Study (KHSS) [12], a population-based face-to-face survey, provides an opportunity to investigate the prevalence and clinical impact of anxiety and depression in individuals with TTH. We hypothesized that 1) prevalence of anxiety and depression would be increased according to headache frequency, 2) demographics, headache characteristics, and associated symptoms would be different according to accompanying of anxiety and depression, 3) headache frequency, headache intensity, and headache impact would be different according to accompanying of anxiety and depression in general population with TTH. For confirming these hypotheses, in this study, we investigated 1) the prevalence of TTH, anxiety, and depression in the general population of Korea; 2) the prevalence of anxiety and depression among participants with TTH; and 3) the differences in clinical characteristics of individuals with TTH, according to their anxiety and depression status using the data of KHSS.

## Materials and Methods

### Survey

We used the data from the KHSS in the current study. The KHSS is a cross-sectional nationwide survey for headaches and symptoms of anxiety and depression among the Korean people aged 19–69 years. Although the study design and methods was described in detail previously [12, 13], we briefly describe as follows. We used a 2-stage clustered random sampling method for most of Korean districts except Jeju-do, and sampled participants proportionally to the population distribution. The 15 administrative divisions were designated as the primary sampling units. Appropriate sample numbers were assigned at each primary sampling unit according to the population distribution. In the second stage, we further selected representative basic administrative units (si, gun, and gu; corresponding state, province, or county in English) for each primary sampling unit. Overall, 60 representative basic administrative units were selected for this study, in each of which we assigned a target sampling number regarding age, gender, and occupation. The estimated sampling error of our study was ± 1.8%, with a 95% confidence interval. We informed participant that the topic of this survey was social health issues rather than headaches to minimize the interest bias. All interviewers were employees of Gallup Korea and had previous experience in social survey. The present study was conducted by door-to-door visits and face-to-face interviews using questionnaire and we only have missing data of our participants regarding the education level. We did not adopt imputation technique during the analysis. Therefore, sensitivity analysis is not needed. Our result was identical to analysis using list-wise deletion method. The study was approved by the institutional review board and ethics committee of Hallym University Sacred Heart Hospital and was performed in accordance with the ethical standards laid out in the 1964 Declaration of Helsinki and its subsequent amendments [14]. Written informed consent was obtained from all participants.

### Diagnosis of TTH

Diagnoses of TTH was based on criteria B through D for infrequent TTH (code 2.1) in the third edition beta version of the International Classification of Headache Disorders (ICHD-3 beta) [B: attack duration from 30 minutes to 7 days; C: at least 2 of the 4 typical headache characteristics (i.e., bilateral location, non-pulsating quality, mild-to-moderate pain intensity, and not aggravated by movement and D: attacks associated with both of the following: no nausea or vomiting and no more than one of photophobia and phonophobia]. Participants who met all of these criteria were considered to have TTH [15]. We did not apply the frequency criterion (criterion A) in the diagnosis of TTH. Thus, the TTH evaluated in this study included infrequent TTH (code 2.1), frequent TTH (code 2.2) and chronic TTH (code 2.3). According to ICHD-3 beta, if a participant’s headache met both the criteria of TTH and probable migraine (PM), we assigned her/him as having TTH [15]. We assessed associated nausea, vomiting, photophobia, phonophobia and osmophobia as accompanying symptoms of headache owing to ICHD-2 and its Appendix, which was the official criteria of headache disorders at our survey [16].

### Diagnosis of anxiety

Anxiety was measured by using the Goldberg Anxiety Scale (GAS) in the present study. The GAS is consisted of four screening items and five supplementary items [17, 18]. Participants who respond positively at two or more screening items and five or more of all of the scale items were diagnosed as having anxiety. The Korean version GAS showed 82.0% sensitivity and 94.4% specificity for the diagnosis of anxiety [18] and previously validated [19].

### Diagnosis of depression

To diagnose depression, the Patient Health Questionnaire-9 was used [19]. Participants who scored 10 or more on this measure were considered to have depression. The Korean version Patient Health Questionnaire-9 showed 81.1% sensitivity and 89.9% specificity [20]. Additionally, to assess headache intensity and impact of headache, we used the visual analogue scale (VAS) and the Headache Impact Test-6 (HIT-6) scores, respectively.

### Analyses

The Kolmogorov—Smirnov test was used to confirm the normality of the distribution; following confirmation, we used the Student’s t-test and Chi-square test for the comparison where appropriate. A significance level of *p* < 0.05 was used for all analyses. Analyses were conducted using the Statistical Package for the Social Sciences 22.0 (SPSS 22.0; IBM, Armonk, NY, USA).

We calculated the odds ratios (OR) and 95% confidence intervals (CI) for the presence of anxiety or depression among participants with TTH compared to non-headache participants by using univariable and multivariable logistic regression analyses. In univariable analyses, we modelled the ORs for participants with TTH versus non-headache participants without adjusting for covariates. In multivariable analyses, anxiety, depression, and sociodemographic variables (age, sex, educational level, and size of the residential area) were used as covariates. In multivariable analyses for depression, sociodemographic variables and anxiety were used as covariates. We analysed the association between mental problems (anxiety and depression) and headache frequency with logistic regression analysis applying the presence of anxiety/depression serving as a dependent variable and headache frequency serving as an independent variable. We compared headache frequency per month, VAS score of headache intensity, and HIT-6 score according to the presence of anxiety and depression using the Mann-Whitney *U* test because of non-normal distributions for these samples. As with most survey sampling designs, there were missing data because of non-responders on several variables. All of the reported findings are based on the available data; as such, the total numbers of some variables differ from the total number of 2695 participants because of missing data. We did not employ imputation techniques because we wanted to minimize non-responding effects [21].

## Results

### Survey

**Fig 1:**
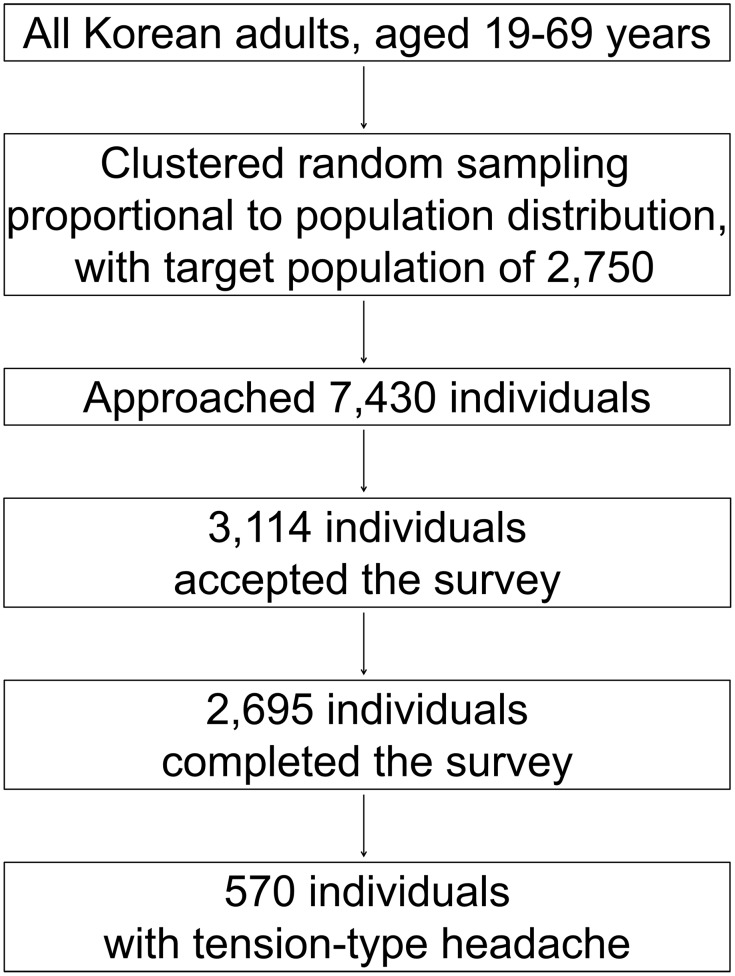
Flow chart depicting the flow of participants in the Korean Headache-Sleep Study.

Our interviewers approached 7,430 individuals and 3,114 of them agreed to the survey (rejection rate of 58.1%). Finally, 2,695 participants completed the survey (cooperation rate of 36.2%)(Fig 1). Distributions of age, gender, educational level and size of the residential area were not significantly different from those of the overall population of Korea (S1 Table) [12]. When comparing sociodemographic variables (age, gender, educational level and size of the residential) between participants with TTH and non-headache participants, female sex and rural area residents demonstrated a higher TTH prevalence compared to male sex and non-rural area residents, respectively. However, the distribution of age and educational level was not significantly different between two groups (Table 1).

**Table 1:** Sociodemographic characteristics of participants with tension-type headache and non-headache participants. Values are presented as number or number (%).

	Tension-type headache participants, N (%)	Non-headache participants, N (%)	*p* value^a^	All survey participants, N^b^
**Sex**			<0.001	8
Male	268 (47.0)	838 (58.9)		1345
Female	302 (53.0)	584 (41.1)		1350
**Age**			0.405	
19–29	119 (20.9)	286 (20.1)		542
30–39	127 (22.3)	293 (20.6)		604
40–49	131 (23.0)	295 (20.7)		611
50–59	107 (18.7)	303 (21.4)		529
60–69	86 (15.1)	245 (17.2)		409
**Size of the residential area**			0.009	
Large city	251 (44.0)	647 (45.5)		1248
Medium-to-small city	243 (42.6)	650 (45.7)		1186
Rural area	76 (13.4)	125 (8.8)		261
**Education level**			0.370	
Middle school or less	96 (16.9)	208 (14.6)		393
High school	247 (43.3)	646 (45.4)		1208
College or more	223 (39.1)	551 (38.7)		1068
Not responded	4 (0.7)	19 (1.3)		26
**Total**	570	1422		2695

^a^: Comparing tension-type headache participants with non-headache participants

^b^: Including tension-type headache participants, non-tension-type headache participants, and non-headache participants.

### Prevalence of TTH, anxiety, and depression

Of the 2,695 survey responders, 1,273 (47.2%) had had at least 1 headache attack during 1 year, and 570 (21.2%) were identified to have TTH in the previous 1 year. Among 570 participants classified as having TTH, 113 (19.8%) of them also met the criteria of PM. Furthermore, two hundred and sixty-eight (9.9%) had anxiety and 116 (4.3%) according to our survey (S2 Table) [22].

### Anxiety and depression in participants with TTH

Among the 570 participants with TTH, 54 (9.5%) had anxiety and 24 (4.2%) had depression. There was an overlap between anxiety and depression among participants with TTH. 44 participants with TTH (7.7%) had anxiety alone, 14 (2.5%) had depression alone, and 10 (1.8%) had both anxiety and depression. The remaining 502 (88.1%) did not have both anxiety and depression. The prevalence of anxiety among participants with TTH (9.5%) was higher than that of non-headache participants (5.3%, OR = 1.9, 95% CI = 1.3–2.7); this pattern remained consistent even after adjusting for sociodemographic variables (age, sex, educational level, and size of the residential area) and depression (OR = 1.7, 95% CI = 1.2–2.5). The prevalence of depression among participants with TTH (4.2%) was higher than that of non-headache participants (1.8%, OR = 2.5, 95% CI = 1.4–4.3); after adjusting for sociodemographic variables and anxiety (OR = 1.9, 95% CI = 1.0–3.5), the OR remained increased.

We further compared the prevalence of anxiety and depression among TTH participants according to fulfilling PM criteria. Participants with TTH fulfilling PM criteria showed a higher prevalence of depression (8.0% vs. 3.3%, *p* = 0.026) compared to those not-fulfilling PM criteria. However, the prevalence of anxiety (14.2% vs. 8.3%, *p* = 0.057) was marginally insignificant between 2 groups. When comparing between TTH participants not-fulfilling PM criteria and non-headache participants, the prevalence of anxiety (8.3% vs. 5.3%, *p* = 0.021) was higher among TTH participants not-fulfilling PM criteria but depression was marginally insignificant (3.3% vs. 1.8%, *p* = 0.050).

### Prevalence of anxiety and depression according to the headache frequency

We noted a significant increase of the prevalence of anxiety among participants with TTH according to the frequency of headache. Participants who experienced 1–14 attacks per month (13.1%, OR = 2.2, 95% CI = 1.2–3.9) and ≥15attacks per month (21.4%, OR = 4.0, 95% CI = 1.0–15.3) had increased ORs compared to those with <1 attack per month (6.4%). However, the prevalence of depression did not significantly differ according to headache frequency among participants with TTH (Table 2).

**Table 2:** Prevalence and odds ratios of anxiety and depression among participants with tension-type headache according to headache frequency. OR: odds ratio, CI: confidence interval.

	Anxiety			Depression		
Headache frequency (per month)	N_case_/N_total_ (%)	OR (95% CI)	*p* value	N_case_/N_total_ (%)	OR (95% CI)	*p* value
**<1**	21/327 (6.4)	Reference	Reference	12/327 (3.7)	Reference	Reference
**1–14**	30/222 (13.1)	2.2 (1.2–3.9)	0.008	11/229 (4.8)	1.3 (0.6–3.1)	0.51
**≥15**	3/14 (21.4)	4.0 (1.0–15.3)	0.004	1/14 (7.1)	2.0 (0.2–16.7)	0.515

For multivariable analysis, sex, age, size of the residential area, and education level were adjusted withpresence of anxiety/depression serving as a dependent variable and headache frequency serving as an independent variable.

### Comparison of clinical characteristics of participants having TTH, with and without anxiety and depression

We investigated the demographics, headache characteristics, headache frequency per month, associated symptoms, VAS score for pain intensity, and HIT-6 score of participants with TTH grouped according to anxiety and depression status. Comparing participants with TTH according to whether participants had anxiety or depression or not, TTH participants who had anxiety or depression showed more frequent phonophobia and osmophobia (S2 Table). Demographics, headache characteristics, and associated symptoms of participants with TTH were not significantly different according to status of anxiety and depression status, except for phonophobia and osmophobia (Table 3-A and 3-B). Headache frequency per month (median, interquartile range (IQR) [1.0, (0.3–2.0) vs. 0.4, (0.2–2.0), *p* = 0.010], VAS score for headache intensity [5.0, (3.0–7.0) vs. 4.0, (3.0–6.0), *p* = 0.010] and HIT-6 [45.5, (42.0–50.3) vs. 42.0, (38.0–47.0), *p* < 0.001] scores were significantly higher with the presence of anxiety (Table 4). VAS scores for headache intensity (median, IQR) [5.0, (3.3–7.0) vs. 4.0, (3.0–6.0), *p* = 0.010] and HIT-6 scores [45.5, (40.5–51.5) vs. 42.0, (40.0–48.0), *p* = 0.027] were significantly higher among TTH participants with depression compared to TTH participants without depression. Headache frequency (median, IQR) [0.7, (0.3–2.8) vs. 0.4, (0.2–2.0), *p* = 0.207] was not significantly different by depression status.

**Table 3:** Demographics, headache characteristics, and associated symptoms of participants with tension-type headache according to of anxiety (A) and depression (B) status. Values are presented as mean ± standard deviation or number (percent). TTH: tension-type headache, SD: standard deviation.

**A**				
		**TTH participants with anxiety (N = 54)**	**TTH participant without anxiety (N = 516)**	***p* value**
**Demographics**				
	Age, years ± SD	42.7 ± 14.4	42.7 ± 13.7	0.969
	Female	35 (64.8)	267 (51.7)	0.067
**Headache characteristics**				
	Bilateral pain	33 (61.1)	341(66.1)	0.464
	Non-pulsating quality	21 (38.9)	206 (39.9)	0.883
	Mild-to-moderate severity	54 (100.0)	506 (98.1)	0.302
	Not aggravated by movement	51 (94.4)	473 (91.7)	0.476
**Accompanying symptoms**				
	Photophobia	3 (5.6)	43 (8.3)	0.476
	Phonophobia	31 (57.4)	150 (29.1)	<0.001
	Osmophobia	14 (25.9)	81 (15.7)	0.055
**B**				
		**TTH participants with depression (N = 24)**	**TTH participants without depression (N = 546)**	***p* value**
**Demographics**				
	Age, years ± SD	40.9 ± 13.0	42.8 ± 13.8	0.512
	Female	12 (50.0)	290 (53.1)	0.765
**Headache characteristics**				
	Bilateral pain	13 (54.2)	361 (66.1)	0.228
	Non-pulsating quality	14 (58.3)	213 (39.0)	0.058
	Mild-to-moderate severity	23 (95.8)	537 (98.4)	0.358
	Not aggravated by movement	19 (79.2)	432 (79.1)	0.996
**Accompanying symptoms**				
	Photophobia	2 (8.1)	44 (8.1)	0.961
	Phonophobia	12 (50.0)	169 (31.0)	0.05
	Osmophobia	9 (37.5)	86 (15.8)	0.005

**Table 4:** Mean headache frequency, headache pain intensity (VAS score), and Headache Impact Test-6 score among participants with tension-type headache according to anxiety and depression status. Values are presented as median (interquartile ranges). VAS: visual analogue scale, HIT-6: Headache Impact Test-6.

	Anxiety			Depression		
	Anxiety (+) Median (25% - 75%)	Anxiety (-) Median (25% - 75%)	*p* value	Depression (+) Median (25% - 75%)	Depression (-) Median (25% - 75%)	*p* value
**Headache frequency (per month)**	1.0 (0.25–2.0)	0.4 (0.2–2.0)	0.01	0.7 (0.3–2.8)	0.4 (0.2–2.0)	0.207
**VAS score**	5.0 (3.0–7.0)	4.0 (3.0–6.0)	0.021	5.0 (3.3–7.0)	4.0 (3.0–6.0)	0.036
**HIT-6 score**	45.5 (42.0–50.3)	42.0 (38.0–47.0)	<0.001	45.5 (40.5–51.5)	42.0 (40.0–48.0)	0.027

## Discussion

The main findings in this study were: 1) the prevalence of anxiety, depression, and TTH in the Korean population was 9.9%, 4.3% and 21.2%, respectively; 2) among participants with TTH, 9.5% had anxiety and 4.2% had depression and these rates were higher than those of non-headache participants; and 3) VAS scores for headache intensity and HIT-6 scores were significantly higher among participants with TTH with anxiety or depression than among those without either condition.

There are some prevalence studies that have shown the 1-year prevalence of TTH, ranged between 35% to 78% in Europe and 20% to 40% in North America [23–25]. In Asian countries, the 1-year TTH prevalence varied between 10.8% and 33.3%, which was somewhat lower than that in European and North American countries [23, 26, 27]. Thus, the 1-year prevalence of TTH in the current study was similar to that of prevalence in Asian countries. Possible explanations for these discrepancies in TTH prevalence between Western and Asian countries are differences in ethnicity, economic status and cultural background.

Previous clinic-based studies of anxiety and depression among participants with TTH showed that a significant proportion of participants with TTH also had anxiety and depression. An Italian study reported that 53.4% and 36.9% of TTH participants had anxiety and depression, respectively [4]. Another clinic-based study in America revealed that the frequency of anxiety and depression among patients with chronic TTH was 17% and 21%, respectively [5]. However, anxiety and depression among participants with TTH using population level data have rarely been reported. In this study, we found that small but a significant proportion of participants with TTH had anxiety and/or depression in this population-based study.

According to the general rule of ICHD-3 beta, we classified 113 participants who met both criteria of TTH and PM as having TTH [15]. The present study demonstrated that the prevalence of anxiety (marginally) and depression among TTH participants fulfilling PM criteria was higher than those not-fulfilling PM criteria. These findings suggested that migrainous features might be associated with a higher prevalence of anxiety or depression even among individuals with TTH. Our study also revealed that approximately 1/5 of participants with TTH fulfilled the criteria of PM in a general population-based sample.

In our study, the prevalence rate of anxiety and depression in the total sample was slightly higher than those of TTH participants. Although we cannot suggest exact cause for these discrepancies, relative high prevalence rate of anxiety and depression of migraine patients could affect our results [22].

Emotional and psychological problems, such as stress, tension, anxiety, and depression have been reported as risk factors for TTH [28]. There are some evidences that chronic TTH headache is associated with frequent and severe stressful life events which may be related with anxiety and depression [29]. In addition, anxiety and depression frequently accompany episodic and chronic TTH [4]. Our study revealed that a small but significant proportion of participants with TTH also had anxiety and depression, which was significantly higher than that of non-headache participants. Furthermore, prevalence of anxiety was increased in participants with more frequent TTH. Therefore, our study reaffirms relationship between psychological disorders (anxiety or depression) and TTH.

We also investigated the prevalence of anxiety and depression according to TTH frequency; notably, anxiety was more prevalent among participants with relatively frequent TTH than among those with infrequent TTH, but this relationship was not present in patients with depression. The higher prevalence of anxiety among participants with frequent TTH supports an association between anxiety and TTH. However, the exact mechanisms responsible for the association with anxiety but not depression are still unknown. Several limitations, such as the small sample size of those with depression in the multivariable analysis, study design, and research methods, may be responsible for these discrepant findings and the cautious interpretation of our results is warranted.

In our study, headache frequency, VAS scores of headache intensity, and HIT-6 scores were significantly higher among TTH participants with anxiety compared to those who without anxiety. Moreover, VAS scores of headache intensity and HIT-6 scores were also higher among TTH participants with depression compared to those without depression. In contrast, mean headache frequency was similar between TTH with depression and those without depression. Because headache frequency is known as a risk factor for the chronification of headache, our results suggest that the presence of anxiety and/or depression among patients with TTH may be associated with its chronification [30]. The increased severity of headache among participants with TTH and anxiety or depression relative to those without either condition suggests a larger or enhanced burden of pain for these comorbid participants. Furthermore, an association of anxiety and depression with an elevated headache severity may be at least partially explained by an emotional and psychological component of pain perception [31]. In a previous multicentre clinical study, patients with headache and anxiety or depression had a significantly diminished quality of life and increased burden such as an unstable employment status, reduced earnings, and less success in their career [11]. Our population-based study is consistent with this prior study [11] and provides additional information about the association between anxiety and/or depression with diminished quality of life among patients with TTH.

Osmophobia and phonophobia seem highly predictive of both anxiety and depression in TTH in our study. The osmophobia and phonophobia are closely associated with migraine. Moreover, these osmophobia and phonophobia consistently showed the positive relationship with presence of anxiety and depression [32, 33]. Considering TTH, both osmophobia (14.5%) and phonophobia (15.9%) are not infrequently reported in previous studies, even though not as much as high comparing that of migraine [32, 34, 35]. In addition, osmophobia may be associated with a higher conversion rate from TTH to migraine [36]. Regarding mechanism for association osmophobia and phonophobia with TTH, osmophobia, phonophobia and psychiatric disorders might possibly result from dysfunction of similar brain structures, such as the insular, hippocampus or amygdala which are related with emotion including anxiety and/or depression [37]. Nevertheless, exact mechanism of relationship osmophobia and phonophobia with TTH accompanying anxiety and/or depression has been still not concluded in our study. Therefore, our results should be carefully interpreted.

In our study, although the response rate seems to be low, we only have missing value on the education level. Therefore, the effect of missing value on the prevalence would be minimal. Moreover, the distribution of sociodemographic variables of our sample was similar to that of Korean general population. In addition, the prevalence of headache and TTH were similar to those of previous studies. Therefore, we could assure that we successfully sample in the present study.

This study had several limitations. First, we diagnosed TTH in face-to-face interviews using a questionnaire. Although the questionnaire was validated by comparing the TTH diagnosis made by neurologists in an additional telephone interview and had high sensitivity and specificity, some participants might have been misdiagnosed [38]. The diagnosis of headache by a physician in an epidemiological study is challenging to achieve. Secondly, although the current study used a population-based sample with low sampling error, its statistical power was limited in terms of examining the subgroups of interest. In other words, the lack of significant findings in the subgroup analyses could be the result of a limited sample size. Third, other important variables, such as employment status, occupation, social class, income, and marital status were not included and adjusted for analysis in our study. Fourth, our study did not adjust chronic pain disorders, such as fibromyalgia. Because chronic pain disorders are important potential confounding factors for psychiatric comorbidities, further study is mandatory for investigating relationship between chronic pain disorders and anxiety and/or depression in headache patients.

Our study has several strengths. First, we used data from the KHSS, which used clustered random sampling proportional to the population distribution with a low sampling error. Secondly, we investigated both anxiety and depression, which are common comorbid conditions both in the general population and among headache sufferers, and assessed the clinical characteristics of TTH with anxiety and/or depression. Balancing these limitations and strengths, we successfully assessed the association and clinical implications of anxiety and depression among patients with TTH.

## Conclusions

Anxiety and depression were present in small but significant proportion of participants with TTH and were associated with an exacerbation of TTH symptoms. Our findings suggest that the proper diagnosis and treatment of anxiety and depression are needed for the improved management of TTH.

## Supporting Information

S1 Table Sociodemographic characteristics of survey participants, the total Korean population, and cases identified as having tension-type headache, anxiety, and depression. (DOC)

S2 Table Demographics, headache characteristics, and associated symptoms between tension-type headache with anxiety or depression and those who without anxiety or depression. (DOCX)
