# Supplementary material for: Anxiety and Depression in Tension-Type Headache: A Population-Based Study
Source: PLoS One. 2016 Oct 26;11(10):e0165316. doi: 10.1371/journal.pone.0165316 (PMC5082613; doi:10.1371/journal.pone.0165316)
Supplement: S1 Table — (DOC) [file pone.0165316.s001.doc]

**S1 Table.** **Sociodemographic characteristics of survey participants, the total Korean population, and cases identified as having tension-type headache, anxiety, and depression.**

|  | | **Survey participants**  **N (%)** | **Total population**  **N (%)** | **p** | **Tension-type headache**  **N, % (95% CI)** | **Anxiety**  **N, % (95% CI)** | **Depression**  **N, % (95% CI)** |
| --- | --- | --- | --- | --- | --- | --- | --- |
| **Sex** | |  |  |  |  |  |  |
|  | Male | 1345 (49.3) | 17,584,365 (50.6) | 0.854^a^ | 268, 19.9 (17.8-22.0) | 109, 8.1 (6.6-9.6) | 43, 3.2 (2.3-4.2) |
|  | Female | 1350 (50.7) | 17,198,350 (49.4) |  | 302, 22.3 (20.1-24.6) | 159, 11.8 (10.1-13.5) | 73, 5.4 (4.2-6.6) |
| **Age** | |  |  |  |  |  |  |
|  | 19–29 | 542 (20.5) | 7,717,947 (22.2) | 0.917^a^ | 119, 22.0 (18.5-25.5) | 53, 9.6 (7.2-12.1) | 23, 4.1 (2.5-5.8) |
|  | 30–39 | 604 (21.9) | 8,349,487 (24.0) |  | 127, 21.0(17.8-24.3) | 51, 8.7 (6.4-11.0) | 32, 5.4 (4.6-7.3) |
|  | 40–49 | 611 (23.1) | 8,613,110 (24.8) |  | 131, 21.4 (18.2-24.7) | 67, 11.0 (8.5-13.5) | 24, 4.0 (2.5-5.5) |
|  | 50–59 | 529 (18.9) | 6,167,505 (17.7) |  | 107, 20.2 (16.8-23.7) | 53, 9.9 (7.3-12.5) | 22, 4.2 (2.5-6.0) |
|  | 60–69 | 409 (15.6) | 3,934,666 (11.3) |  | 86, 21.0 (17.1-25.0) | 44, 10.8 (7.8-13.8) | 15, 3.7 (2.0-5.5) |
| **Size of the residential area** | |  |  |  |  |  |  |
|  | Large city | 1248 (46.3) | 16,776,771 (48.2) | 0.921^a^ | 251, 20.1 (17.9-22.4) | 130, 10.4 (8.7-12.1) | 57, 4.6 (3.4-5.7) |
|  | Medium-to-small city | 1186 (44.0) | 15,164,345 (43.6) |  | 243, 20.5 (18.2-22.8) | 112, 9.5 (7.8-11.2) | 47, 4.0 (2.9-5.1) |
|  | Rural area | 261 (9.7) | 2,841,599 (8.2) |  | 76, 29.1 (23.6-34.7) | 26, 10.0 (6.3-13.6) | 12, 4.7 (2.1-7.3) |
| **Education level** | |  |  |  |  |  |  |
|  | Middle school or less | 393 (14.9) | 6,608,716 (19.0) | 0.752^a^ | 96, 24.5 (20.1-28.7) | 55, 13.9 (10.5-17.4) | 20, 5.2 (3.0-7.4) |
|  | High school | 1208 (44.5) | 15,234,829 (43.8) |  | 247, 20.5 (18.2-22.7) | 111, 9.2 (7.5-10.8) | 49, 4.1 (3.0-5.2) |
|  | College or more | 1068 (39.6) | 12,939,170 (37.2) |  | 223, 20.9 (18.4-23.3) | 100, 9.5 (7.7-11.2) | 47, 4.4 (3.2-5.7) |
|  | Not responded | 26 (9.6) |  |  | 4, 15.4 (0.5-30.2) | 2, 8.0 (0.0-18.0) | 0, 0.0 (0.0-0.0) |
| **Total** | | 2695 (100.0) | 34,782,715 (100.0) |  | 570, 21.2 (19.6-22.7) | 268, 10.0 (8.8-11.1) | 116, 4.3 (3.6-5.1) |

N: number, CI: confidence interval.

Values are presented as number (%) or number, % (95% confidence interval).

^a^: Sex, age group, size of the residential area, and education level was compared between the sample of the present study and the total population of Korea.
