# Supplementary material for: Anxiety and Depression in Tension-Type Headache: A Population-Based Study
Source: PLoS One. 2016 Oct 26;11(10):e0165316. doi: 10.1371/journal.pone.0165316 (PMC5082613; doi:10.1371/journal.pone.0165316)
Supplement: S2 Table — (DOCX) [file pone.0165316.s002.docx]

**S2 Table.** **Demographics, headache characteristics, and associated symptoms between tension-type headache with anxiety or depression and those who without anxiety or depression.**

|  |  | **TTH participants with anxiety or depression (N = 68)** | **TTH participant without anxiety or depression (N = 502)** | ***p* value** |
| --- | --- | --- | --- | --- |
| **Demographics** |  |  |  |  |
|  | Age, years ± SD | 42.6 ± 14.2 | 42.7 ± 13.6 | 0.979 |
|  | Female | 39 (57.4) | 263 (52.4) | 0.518 |
| **Headache characteristics** |  |  |  |  |
|  | Bilateral pain | 42 (61.8) | 332 (66.1) | 0.498 |
|  | Non-pulsating quality | 29 (42.6) | 198 (39.4) | 0.692 |
|  | Mild-to-moderate severity | 67 (98.5) | 496 (98.8) | 0.591 |
|  | Not aggravated by movement | 50 (73.5) | 401 (79.9) | 0.265 |
| **Accompanying symptoms** |  |  |  |  |
|  | Photophobia | 5 (7.4) | 41 (8.2) | 1 |
|  | Phonophobia | 36 (52.9) | 145 (28.9) | <0.001 |
|  | Osmophobia | 18 (26.5) | 77 (15.3) | 0.036 |

Values are presented as mean ± standard deviation or number (percent).

TTH: tension-type headache, SD: standard deviation
